# Solar-driven butanedione-based disinfection: A by-product-free approach to pathogen control

**DOI:** 10.1016/j.fmre.2025.03.003

**Published:** 2025-03-14

**Authors:** Zewen Ji, Wenchang Zhang, Jianghua Yang, Peng Peng, Haoran Chen, Shujuan Zhang

**Affiliations:** State Key Laboratory of Pollution Control and Resource Reuse, School of the Environment, Nanjing University, Nanjing 210023, China

**Keywords:** Bacterial inactivation, Diketone, Acetyl peroxyl radical, Metabolomics, Disinfection by-products (DBPs)

## Abstract

The imperative for environmentally friendly water disinfection methods has spurred the exploration of innovative solutions to mitigate microbial threats in water while simultaneously controlling the formation of harmful disinfection by-products (DBPs). This study introduces the combination of butanedione (BD) and solar irradiation as an effective method to inactivate pathogenic bacteria, including Gram-negative *Escherichia coli* and Gram-positive *Staphylococcus aureus*. Acetyl radicals produced by solar activation of BD homolytic cleavage combined with dissolved oxygen to form acetyl peroxyl radicals, which were the main reactive species in the disinfection process. These acetyl peroxyl radicals attack bacterial cell membranes, escalate intracellular reactive oxygen species, and induce oxidative degradation of vital intracellular components such as enzymes and nucleic acids. Remarkably, the BD/Solar system demonstrated exceptional disinfection efficacy across a range of water quality parameters, achieving rate constants that were 5.2–98.1 times greater than those of other advanced disinfection processes that utilize H_2_O_2_, peroxydisulfate, or periodate in combination with solar irradiation. Notably, there was no evidence of bacterial regrowth and no formation of typical halogenated DBPs after treatment. These findings highlight the potential of this novel solar-powered system as a green approach for controlling environmental pathogens in a variety of waters, especially in sunlit swimming pools.

## Highlights

• BD/Solar system effectively inactivated bacteria in a range of waters.
• Acetyl peroxyl radicals contributed to bacteria inactivation in BD/Solar system.
• BD/Solar inactivated bacteria via physiological and metabolic changes.
• Disinfection with BD/Solar did not generate halogenated disinfection by-products.

## Graphical abstract

**Figure:**
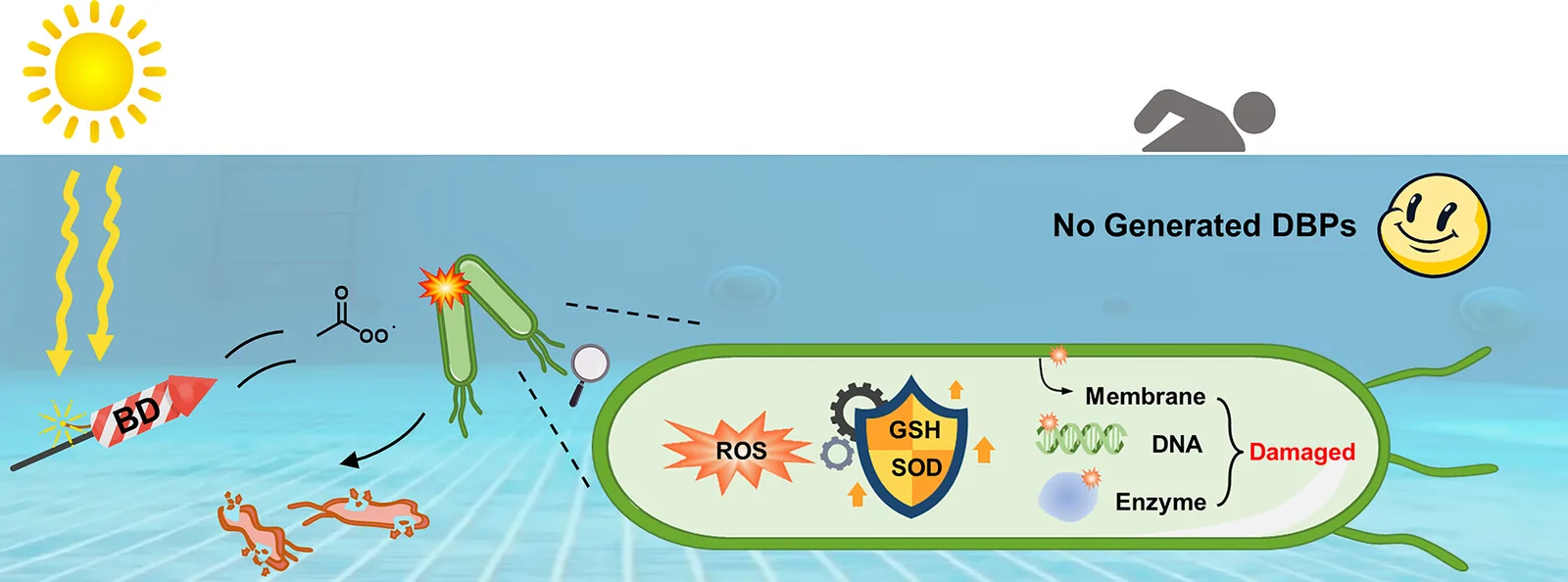

## 1 Introduction

The imperative for effective disinfection strategies is underscored by the global need to control waterborne pathogens and prevent the spread of infectious diseases [1]. Conventional disinfection methods, such as chlorination and ozonation, have a longstanding history but are increasingly scrutinized due to the generation of carcinogenic disinfection by-products (DBPs) and potential health risks associated with direct exposure to these oxidative agents [2,3]. Therefore, there is an urgent need to develop safer and greener technologies for bacterial inactivation.

A variety of advanced oxidation processes (AOPs), leveraging the activation of common oxidants such as hydrogen peroxide (H_2_O_2_), peroxydisulfate (PDS), peroxyacetic acid (PAA), and periodate (PI) to highly reactive oxygen species (ROS), such as hydroxyl radical (•OH), sulfate radical (•SO_4_^–^), superoxide radical anion (•O_2_^–^), and singlet oxygen (^1^O_2_), have been developed and shown excellent efficiency in bacterial inactivation [[4], [5], [6], [7]]. These AOPs are typically activated by energy-intensive methods like UV irradiation or electricity [8], or through the use of costly non-homogeneous catalysts [9], which may introduce secondary contamination through metal ion leaching. Consequently, there is a growing interest in AOPs activation mechanisms that circumvent the need for external energy inputs and avoid the use of metal-based catalysts.

Sunlight provides an ideal and inexhaustible source of energy. Recently, the use of sunlight for water disinfection has garnered extensive attention [10]. However, the challenge lies in the limited activation of many oxidants by visible light (Vis), which accounts for approximately 48% of the sunlight energy [11]. Note that most of the aforementioned oxidants can only be activated under UV irradiation, which accounts for only 5% of the sunlight spectrum. Recent efforts have focused on identifying photo-active chemicals responsive to visible light [12]. While organic photosensitizers such as erythrosine [13], natural extracts [14], and dissolved organic matter (DOM) [15] have been explored for this purpose, their complex photochemical behaviors and potential risks associated with their degradation products necessitate the consideration of safer alternatives [16].

As compared with the above photosensitizers, butanedione (BD), a naturally occurring metabolite with a well-defined photochemical process, offers a promising solution. Widely present in food, vegetables, and the atmosphere, BD is generally considered safe by the United States Department of Agriculture and is categorized as non-hazardous to the aquatic environment by the European Chemicals Agency [17]. Recognized for its antimicrobial properties and safety as a food preservative and flavoring agent [[18], [19], [20]], BD has been used in the food industry for a long history. For example, its concentration ranges from 0.5 to 20 mg/kg in fermented dairy products and 44.0–62.5 mg/kg in bakery products [19]. In recent years, BD has attracted research interests in the environmental field due to its ability to transform a variety of pollutants under UV irradiation [21,22]. Once excited, BD can undergo Norrish cleavage to generate free radicals [23]. Owing to the conjugation and resonance effects of the two adjacent carbonyl groups, BD can absorb and utilize visible light [24]. Given this, activation of BD by sunlight is expected to have the potential to become a safer and environmentally friendly water disinfection technology to overcome the drawbacks of traditional disinfection methods. It is worth mentioning that while previous research has explored solar-based pretreatments [[12], [13], [14], [15]] for pathogen inactivation, none has specifically examined the BD/Solar system as a disinfection method for water.

In this study, BD/Solar was investigated as a disinfection process for the first time. The efficiency of the BD/Solar process for water disinfection was evaluated under a variety of conditions. The photochemical mechanism of this disinfection process was discussed based on some physiological indices, such as antioxidant enzyme activities, intracellular ROS level, oxidation products, and membrane integrity, as well as untargeted metabolomics. Moreover, the production of DBPs in a sunlight-simulated swimming pool and post-disinfection bacterial regeneration were examined to assess the feasibility of the BD/Solar process. The results demonstrate that BD/Solar is a promising technique for bacterial disinfection, paving the way for more efficient and safer water disinfection alternatives.

## 2 Materials and methods

### 2.1 Chemicals and bacteria

Details for the chemicals used are available in Text S1 (online). Ultrapure water, tap water, Jiuxiang River water, and Tianlai Lake water (collected from Nanjing, China) were employed for bacterial inactivation tests. A simulated swimming pool water was also prepared and employed for the detection of DBPs. The water samples were filtered using a 0.22 μm membrane immediately after collection and stored in a refrigerator at 4°C. The water quality parameters of the tested natural water samples are listed in Table S1 (online).

*Escherichia coli* (*E. coli*) and *Staphylococcus aureus* (*S. aureus*) are common pathogens in polluted water and are widely used as model organisms to investigate the physiological and metabolic processes of bacteria. Here, *E. coli* DH5α and *S. aureus* ATCC25923 were selected as model bacteria to carry out the inactivation experiments. Bacterial cells were cultured in LB broth medium at 37°C with shaking at 200 rpm for 12 h (*E. coli*) or 18 h (*S. aureus*) to reach the late exponential phase of growth. The bacteria collected in this phase of growth have more active metabolism, and their physiological processes are stable, allowing for a more accurate assessment of bacterial inactivation properties of disinfectants. The culture solution was centrifuged at 5000 rpm for 5 min to collect the bacterial cells, which were then washed three times with sterilized saline (0.9% NaCl) and re-suspended in sterilized saline to obtain a stock cell suspension, which was diluted and used for subsequent experiments.

### 2.2 Disinfection experiments

The disinfection experiments were conducted with two light sources, Solar and Vis-LED (Fig. S1 online). The irradiation intensities of sunlight and the Vis-LED were measured as 70 mW/cm^2^ and 50 mW/cm^2^, respectively, using an optical power meter (PL-MW2000, PerfectLight, China). While there were differences in the emission spectra and intensities of these two light sources, the photochemical reactions of BD are likely to be consistent. This is because the photolysis of BD is primarily governed by its absorption spectrum rather than the emission spectra of the light sources. Consequently, despite the variations in light source characteristics, the fundamental photochemical behavior of BD during the disinfection process remains reliable. Unless otherwise stated, before irradiation, the stock solution of BD was added to the bacterial suspension to an initial concentration of 0.2 mM, and the initial pH of the tested suspensions was adjusted to 7 using HCl or NaOH. The reaction suspension (1 mL) was taken at certain time intervals during irradiation and the density of culturable bacteria was determined by the plate counting method with a level of detection (LOD) of 10 CFU/mL. More details for the experimental setup are presented in Text S2 (online). The disinfection kinetics were fitted by the Hom model [7]. Detailed reasons for choosing the Hom model with *n* = 2 to describe the disinfection kinetics were provided in Text S3 and Fig. S2 (online).

### 2.3 Analytic methods

The role of active species in the BD/Solar process was explored by quenching experiments with a set of scavengers and trapping experiments with DMPO as a probing molecule. The signals were recorded with an electron spin resonance (ESR) spectrometer (Bruker EMX/Plus).

BD concentration was determined using o-phenylenediamine derivatization combined with high-performance liquid chromatography (HPLC, Ultimate 3000, Dionex) [21]. The lysis of *E. coli* cells was observed with confocal laser scanning microscopy (ALR-SI, Nikon) after staining with the LIVE/DEAD BacLight Bacterial Viability Kit (L13152, Thermo Fisher). The bacterial precipitates were fixed with 2.5% glutaraldehyde and dehydrated with ethanol, and then the morphological changes of *E. coli* cells were observed using scanning electron microscopy (SEM, Zeiss).

The details for ESR and cell component analysis, including superoxide dismutase (SOD), malondialdehyde (MDA), 8-hydroxydeoxyguanosine (8-OHdG), the gene encoding *β*-galactosidase (*lacZ*), K^+^, glutathione (GSH), and adenosine triphosphate (ATP) concentration are available in Text S4 (online). The methods for metabolite extraction and identification are presented in Text S5 (online). To determine the irreversible inactivation of the bacteria, the bacterial suspension was added to the LB medium for re-incubation, and bacterial growth was monitored by absorbance at 600 nm.

### 2.4 DBPs formation

The generation of DBPs was examined in simulated swimming pool water under sunlight exposure. The simulated swimming pool water was prepared by diluting the artificial body fluids (Table S2 online) with tap water (the dilution ratio was about 2500) to an initial total organic carbon (TOC) concentration of about 3 mg/L [25]. The simulated water was then buffered with 5 mM phosphate (pH 7) and placed under sunlight irradiation for 8 h after the addition of NaClO or BD. Thereafter, the concentration of free chlorine was measured using a photometer (HI96701, Hanna) based on the DPD (N, N‑diethyl-p-phenylenediamine) colorimetric method according to the EPA Method 330.5 [26]. The water samples were pretreated, and then the concentrations of several typical DBPs, including trihalomethanes (THMs), haloketones (HKs), haloacetonitriles (HANs), and haloacetic acids (HAAs), were determined with a gas chromatograph (GC-ECD, 7890A, Agilent) [27,28]. More details on DBP detection are available in Text S6, Tables S3-S4 and Fig. S3 online.

### 2.5 Data processing

All experiments were conducted in triplicate, excluding the metabolomics analysis, which was performed six times for biological replicates. The data were shown as a mean ± standard deviation (the error bars in the plots). SPSS 24.0 (SPSS Inc., Chicago, IL, USA) was utilized for the student's *t*-test of the statistical analysis, and the statistical analysis was based on a confidence level of ≥ 95% (*p* < 0.05 was considered statistically significant).

## 3 Results and discussion

### 3.1 Performance of BD/solar for bacterial inactivation

BD is known as an antimicrobial agent [18]. However, as illustrated in Fig. 1a, the number of culturable *E. coli* was not changed after incubation with 0.2 mM BD for 30 min in darkness, indicating that the dosage of BD used in this test was well below the minimum inhibition concentration (MIC) [18,19]. Under otherwise identical conditions, the suspensions exposed to sunlight (Solar) showed observable inactivation. Viable *E. coli* of an initial cell density of 1 × 10^7^ CFU/mL could be inactivated by BD/Solar to below the LOD in 15 min at a rate constant (*k*_obs_) of (31.4 ± 0.3) × 10^–3^ min^-2^ (Fig. 1b). PI/Solar, as reported [29,30], was also effective, but approximately 2 logs of cells remained in the PI/Solar system even after 30 min of irradiation. The *k*_obs_ in the PI/Solar ((6.06 ± 0.01) × 10^–3^ min^-2^) was only about 20% of the BD/Solar counterpart. In comparison, PDS and H_2_O_2_ were significantly less effective. These results demonstrate that BD is a more potent solar disinfectant than the other reported agents. Compared with other AOP-based disinfection systems, the bacterial disinfection efficiency and the cost of the BD/Solar system were competitive (Table S5 online).

**Fig. 1:**
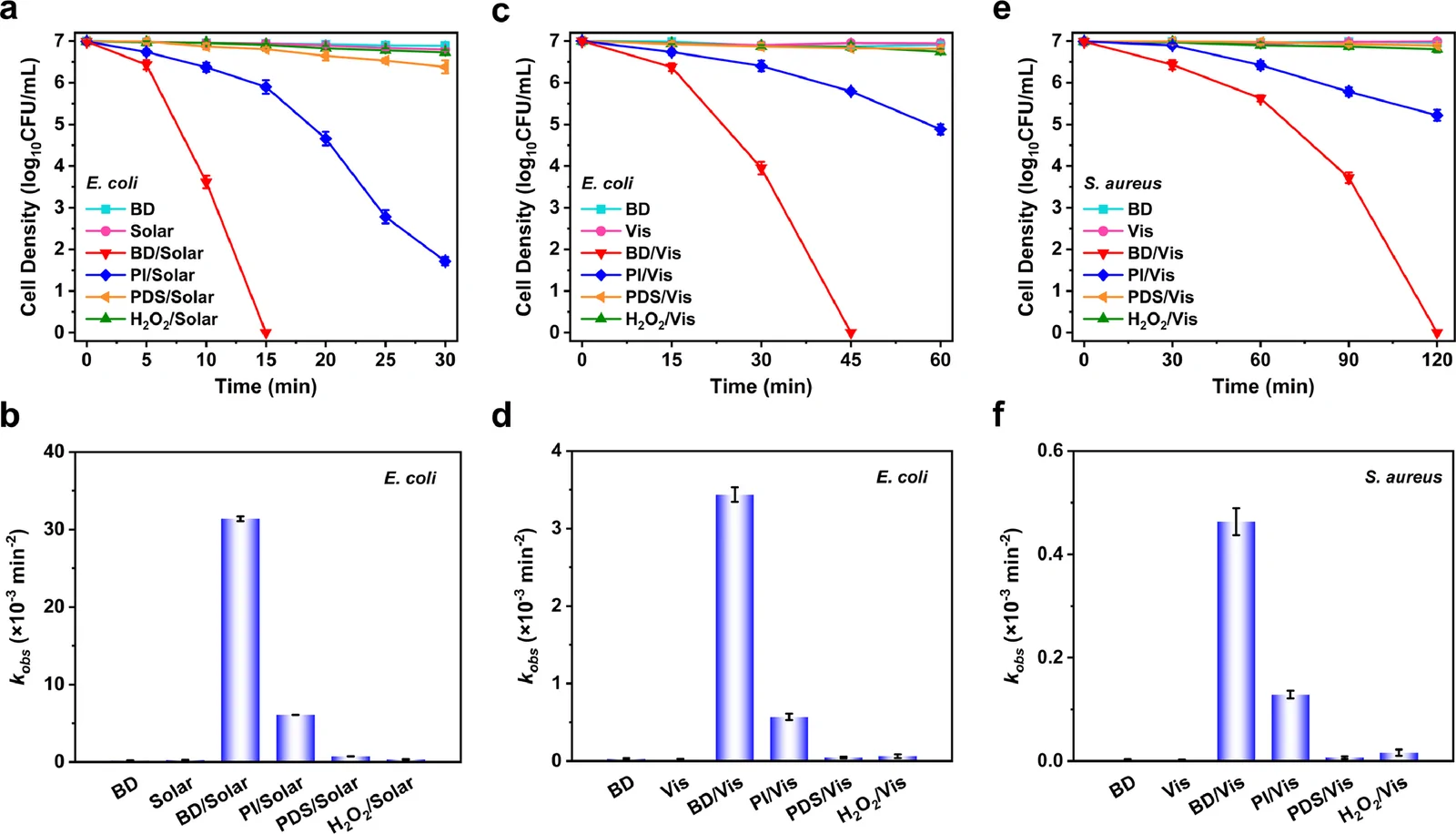
**Disinfection kinetic profiles and observed inactivation rate constants of *E. coli* (a-d) and *S. aureus* (e, f) under various conditions.** Initial cell density: 1 × 10^7^ CFU/mL, [BD]_0_: 0.2 mM.

Considering the uncontrollable variation in the light intensity of natural sunlight, we conducted the following irradiation experiments with Vis-LED to ensure comparability. Under the given light intensity, Vis alone, as expected, was not effective in the inactivation of *E. coli*. In contrast, with the combination of 0.2 mM BD and Vis, the *E. coli* in the suspension were completely inactivated within 45 min (Fig. 1c). The *k*_obs_ of the BD/Vis process ((3.4 ± 0.1) × 10^–3^ min^-2^) was two orders of magnitude higher than those of BD alone ((0.025 ± 0.006) × 10^–3^ min^-2^) or Vis alone ((0.023 ± 0.006) × 10^–3^ min^-2^) (Fig. 1d). More importantly, under otherwise identical conditions, the other common oxidants, including PDS, H_2_O_2_, and PI, only led to a slight *E. coli* inactivation, with *k*_obs_ values of (0.044 ± 0.011) × 10^–3^, (0.062 ± 0.026) × 10^–3^, and (0.57 ± 0.04) × 10^–3^ min^-2^, respectively. The observed efficiencies of the above three oxidants were significantly lower than the reported data in the literature [11,29,31], which might be due to the differences in experimental conditions, including the light source and oxidant dosage. As illustrated in Fig. S1 (online), there was almost no overlap between the absorption spectra of the three common oxidants and the emission spectrum of the light source. Among the four tested chemicals, only BD can utilize the light emitted from the Vis-LED.

In addition to Gram-negative *E. coli*, the performance of BD for disinfection was also investigated with Gram-positive *S. aureus*. Similar to the case of *E. coli*, neither Vis irradiation alone nor BD under dark was effective in inactivating *S. aureus* (Fig. 1e). Excitingly, BD/Vis accomplished complete inactivation of *S. aureus* at an initial cell density of 1 × 10^7^ CFU mL^-1^ in less than 120 min. Notably, the disinfection rate of *S. aureus* (*k*_obs_ = (0.46 ± 0.03) × 10^–3^ min^-2^) was much lower than that of *E. coli* (*k*_obs_ = (3.4 ± 0.1) × 10^–3^ min^-2^) (Fig. 1f). This is because Gram-positive bacteria have thicker and more complex cell wall structures and are more resistant to disinfectants [32,33]. Similar to the case of *E. coli*, the inactivation of *S. aureus* by PDS, H_2_O_2_, and PI was significantly weaker than that of BD under otherwise identical disinfection conditions.

The performance of BD/Solar was also investigated under various conditions (Texts S2 and S7 online). The results indicate that BD/Solar performed well over a wide range of initial BD concentration (0.1 to 0.3 mM), cell density (1 × 10^6^ to 1 × 10^8^ CFU/mL), pH (3 to 9), humic acid concentration (0.1 to 10 mg/L), and co-existing salts (15 mM) at the cost of low BD consumption (Fig. S4-S6 online). Prior to real-world applications, the potential for coupling BD/Solar with other processes should be explored further, e.g. to improve disinfection efficiency by reducing organic matter concentration or turbidity.

### 3.2 Physiology of bacteria during the BD/Solar process

Confocal laser scanning microscopy (CLSM), enhanced by fluorescent dye staining, enables the detection of cell viability and cell membrane integrity. Prior to disinfection, all the bacterial cells exhibited green fluorescence (Fig. 2a), indicating that their membranes were intact. After 15 min treatment with BD/Vis, a small portion of the cells showed red fluorescent, while about 73% of the cells (based on multiple images) still showed green color (Fig. 2b). When the disinfection process was carried out for 75 min, all the bacterial cells showed red fluorescence (Fig. 2c), which was consistent with the complete inactivation of *E. coli* observed in Fig. 1. The SEM observations further verified the above conclusion. The surface of *E. coli* was smooth and intact before the disinfection process (Fig. 2d). After 15 min of treatment with BD/Vis, the cells gradually shriveled, and the surface became concave (Fig. 2e). Eventually, at 75 min, the bacteria completely ruptured and died (Fig. 2f).

**Fig. 2:**
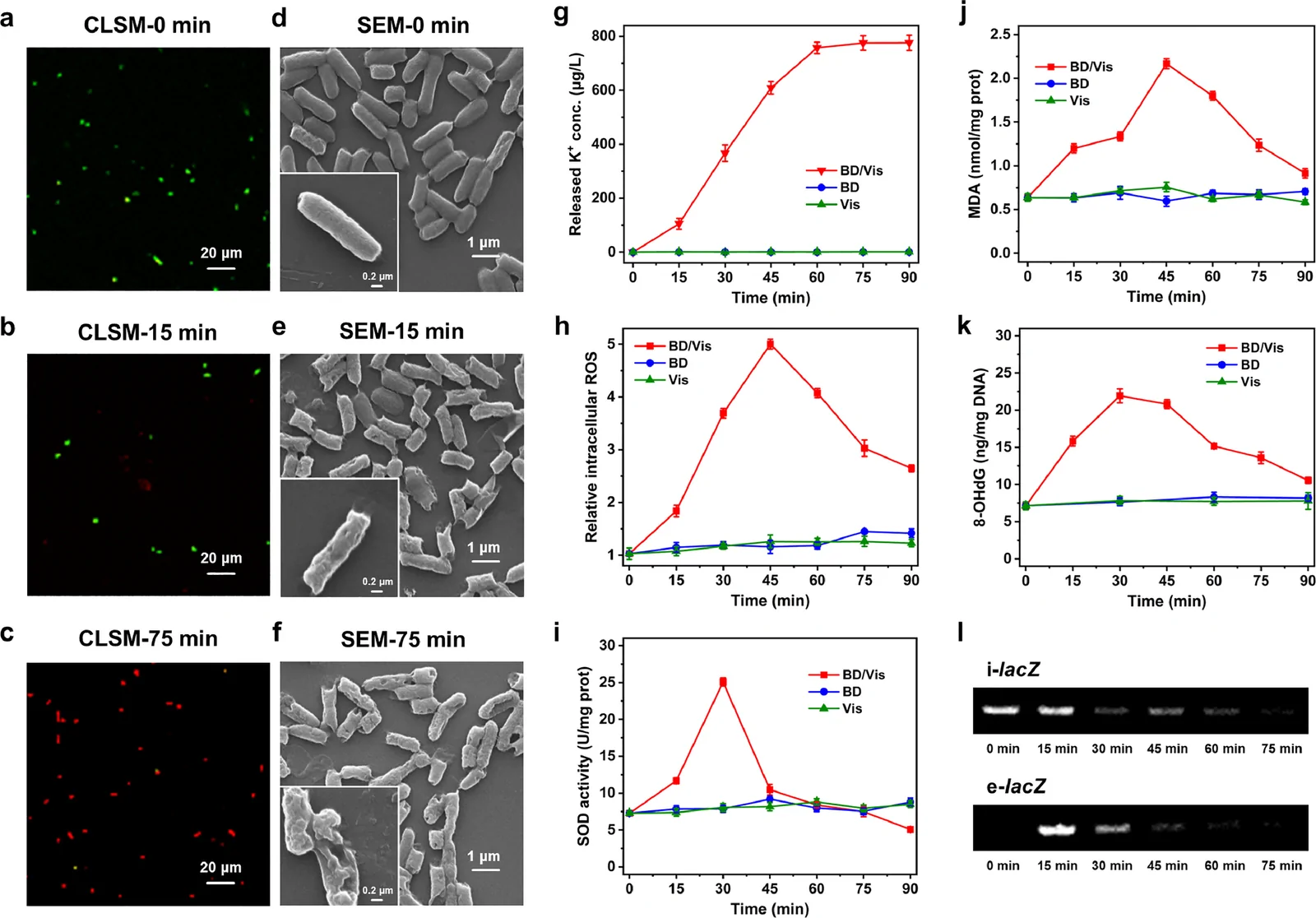
(a–c) Confocal laser scanning microscopic images and (d–f) SEM images of *E. coli* sampled at different disinfection times. (g-k) Concentration of released K^+^, intracellular ROS levels, SOD activity, MDA, and 8-OHdG concentration during the disinfection process. (l) PCR images of *lacZ* genes during disinfection. Initial cell density: 1 × 10^8^ CFU/mL, [BD]_0_: 0.2 mM.

The change in K^+^ concentration in the solution followed a sigmoidal curve (Fig. 2g). Considering that in the late stages of disinfection, most bacteria were killed but the concentration of K^+^ continued to rise, we infer that the outflow of K^+^ was derived from the rupture of dead cells. Correspondingly, as the disinfection process proceeded, the intracellular ROS level increased significantly to five times the initial level within 45 min (Fig. 2h). With a further extension of the treatment time, the ROS level gradually decreased, apparently due to the rupture of the cells [30]. In contrast, no significant changes in ROS levels were observed for the samples with BD or Vis alone.

Bacteria have their antioxidant defense system, the most important of which are intracellular antioxidant proteins like SOD. In the BD/Vis system, the SOD activity increased rapidly from 7.5 U/mg prot to 25 U/mg prot within the first 30 min (Fig. 2i). Thereafter, the SOD activity declined as the treatment time increased, possibly due to the collapsed bacterial defense overwhelmed by the excessive ROS accumulation [34]. It is worth noting that the change in SOD activity closely resembled the process of bacterial inactivation, suggesting that the penetration of ROS into the cytoplasm may be the main driving force behind inactivation.

Accompanied by the accumulation of ROS and the collapse of the oxidation defense system, two typical oxidation products were observed: MDA, the product of cellular lipid peroxidation, which is usually associated with oxidative damage to cell membranes [32], and 8-OHdG, a marker of oxidative DNA damage [35]. The evolution patterns of MDA (Fig. 2j) and 8-OHdG (Fig. 2k) matched well with those of ROS and SOD, respectively. The concentration of 8-OHdG peaked at 30 min at a level of 21.9 ng/mg of DNA, which was 3.1 times higher than the initial level.

DNA damage and gene function are closely interconnected. The gene encoding *β*-galactosidase, also known as *lacZ*, is part of the lac operon in *E. coli*, which is commonly used as a reporter gene in the analysis of cells. At the onset of the BD/Vis treatment, the extracellular *lacZ* gene (*e-lacZ*) exhibited low abundance, while the intracellular *lacZ* gene (*i-lacZ*) showed a significant level of abundance. Throughout the BD/Vis treatment, the abundance of *e-lacZ* initially increased within the first 15 min and then gradually declined. A similar trend was observed for *i-lacZ* (Fig. 2l). These findings suggest cell rupture and degradation of the released genes. It should be noted that only severe damage to chromosomal DNA is lethal to the cell [36]. Otherwise, the bacteria can repair damaged structures and may still recover under suitable conditions, posing potential health risks in the water [37].

The possibility of bacterial revival after BD/Vis treatment was excluded. As illustrated in Fig. S7 (online), the growth profiles of the *E. coli* treated with BD or Vis alone were similar to that of the positive control (original bacterial broth). Their OD_600_ increased rapidly to about 1.1 after the first day of incubation, then slowly increased to about 1.4 within 3 days, and finally decreased to 1.2 within 3–5 days. This phenomenon is consistent with the observation that neither BD nor Vis alone inactivated the bacteria (Fig. 1c). Reassuringly, the OD_600_ of the BD/Vis system mirrored that of the blank control (sterilized saline), which remained unchanged at 0 for 5 days. These results demonstrate that the *E. coli* cells were completely nonviable after the BD/Vis treatment. No regeneration occurred, indicating the absence of viable but non-culturable (VBNC) state bacteria [38]. Due to the low metabolic levels, VBNC bacteria may be less responsive to disinfection methods that rely on cellular metabolism or active growth. Future research could explore the combined use of BD/Vis with other disinfection methods to improve inactivation of VBNC bacteria.

### 3.3 Reactive species involved in the BD/solar process for bacterial inactivation

The results of the physiological analysis indicated oxidative damage to the treated bacteria. The reactive species that accounted for oxidative damage were identified with both trapping and scavenging experiments. The ESR spectra (Fig. 3a), acquired in an oxygen-free environment, predominantly exhibited six major peaks that correspond to the DMPO—CH_3_C(O)• adduct signal [24]. Additional minor peaks, indicative of DMPO—CH_3_•, were also detected. Dissolved oxygen (DO) was found to be pivotal in the BD/Solar system. A significant reduction in the inactivation rate was observed when the concentration of DO was minimized by purging with N_2_, with the *k*_obs_ dropping to (0.19 ± 0.02) × 10^–3^ min^-2^) (Fig. 3b and S8a online).

**Fig. 3:**
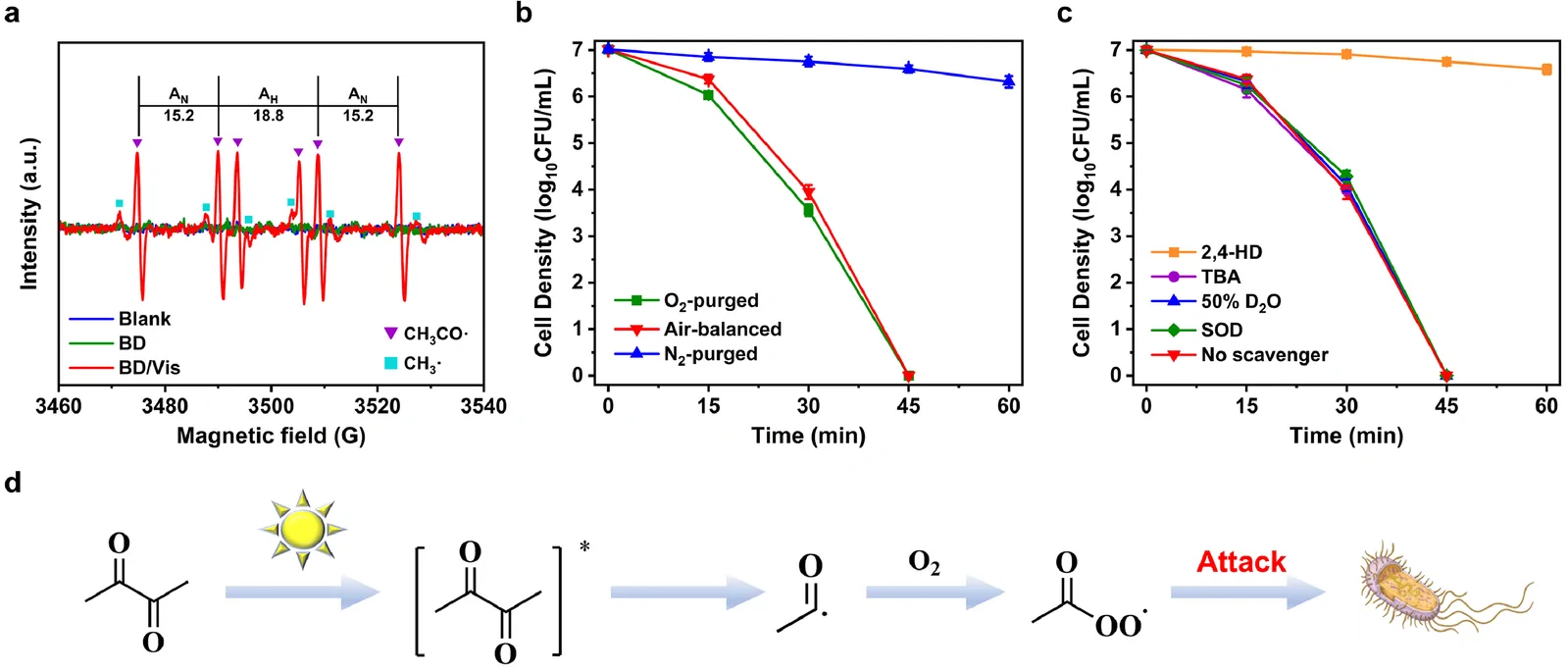
(a) ESR spectra of Vis irradiated BD solution (free of dissolved oxygen). (b, c) Effect of aeration conditions and scavengers on the disinfection performance of BD/Vis for *E. coli*. (d) The schematic illustration of the photochemical reactions in the BD system. Initial cell density: 1 × 10^7^ CFU/mL, [BD]_0_: 0.2 mM, [2,4-HD]: 10 mM, [TBA]: 10 mM, [SOD]: 50 U/mL.

The addition of tert‑butyl alcohol (TBA), a scavenger of hydroxyl radicals (•OH), and SOD, a scavenger of superoxide radicals (•O_2_**^–^**), did not markedly alter the inactivation kinetics of *E. coli* (Fig. 3c and S8b online), suggesting these ROS played a minor role in the disinfection process. The half-life of singlet oxygen (^1^O_2_) is known to be longer in deuterium oxide (D_2_O) compared to regular water (H_2_O) [29]. If ^1^O_2_ were a major active species, substituting H_2_O with D_2_O would enhance the disinfection efficacy. However, no significant enhancement was observed in the BD/Solar system when 50% D_2_O was used to replace H_2_O, indicating that ^1^O_2_ was not a key contributor to the disinfection in the BD/Solar process. Contrastingly, when 2,4-hexadiene (2,4-HD), a scavenger of •OH and alkoxyl radicals (RO•) [39], was introduced to the system, there was a marked decrease in disinfection performance, with the *k*_obs_ value falling to (0.12 ± 0.01) × 10^–3^ min^-2^. This suggests that RO• was the principal active species in the BD/Solar system.

Note that the potential toxic effects of the four scavenging agents were considered and ruled out, as demonstrated in Fig. S9 (online). Thus, the active species generated in the BD/Solar system is proposed in Fig. 3d. Upon solar or Vis-LED irradiation, BD was excited to a higher energy state (BD*). This excited BD then underwent Norrish Type I reaction, which involved the cleavage of the C—C bond adjacent to the carbonyl group, predominantly yielding acetyl radicals (CH_3_C(O)•). CH_3_C(O)• reacted rapidly with DO at a rate constant close to diffusion control to form peroxyl radicals (CH_3_C(O)OO•), which is known as the most oxidative organic radical (*E_0_* = 0.95 V) [21]. Recall that N_2_ purging or 2,4-HD dosing almost completely inhibited the inactivation, CH_3_C(O)OO• might play the most critical role in the BD/Solar disinfection. This is supported by previous reports, in which the CH_3_C(O)OO• radicals came from UV/PAA [40,41]. Compared to other oxidative radicals, CH_3_C(O)OO• has a moderate reduction potential and therefore is more selective. It tends to react with electron-rich groups [21], and is expected to oxidatively destroy biomolecules.

### 3.4 Mechanism of the BD/Solar process for bacterial inactivation

To elucidate the inactivation mechanism, the metabolite profiles of *E. coli* treated with BD/Vis for 30 min (BD group) were compared to those of untreated controls (CK group) using principal component analysis (PCA) (Fig. S10 online). The PCA results revealed distinct clustering between the two groups, indicating that the disinfection process significantly altered the bacterial metabolome.

Differential metabolites were screened and identified by setting a significance threshold (VIP > 1, *p* < 0.05, and fold change (FC) ≥1.2 or ≤0.8). Post disinfection, a total of 129 differential metabolites were observed: 50 were up-regulated, and 79 were down-regulated (Fig. S11a online). These identified differential metabolites were enriched and analyzed using the KEGG database. The results showed that the differential metabolites were mainly associated with nucleotide metabolism, purine metabolism, TCA cycle, oxidative phosphorylation, amino acid metabolism, and glutathione metabolism (Fig. 4 and Text S8 online).

**Fig. 4:**
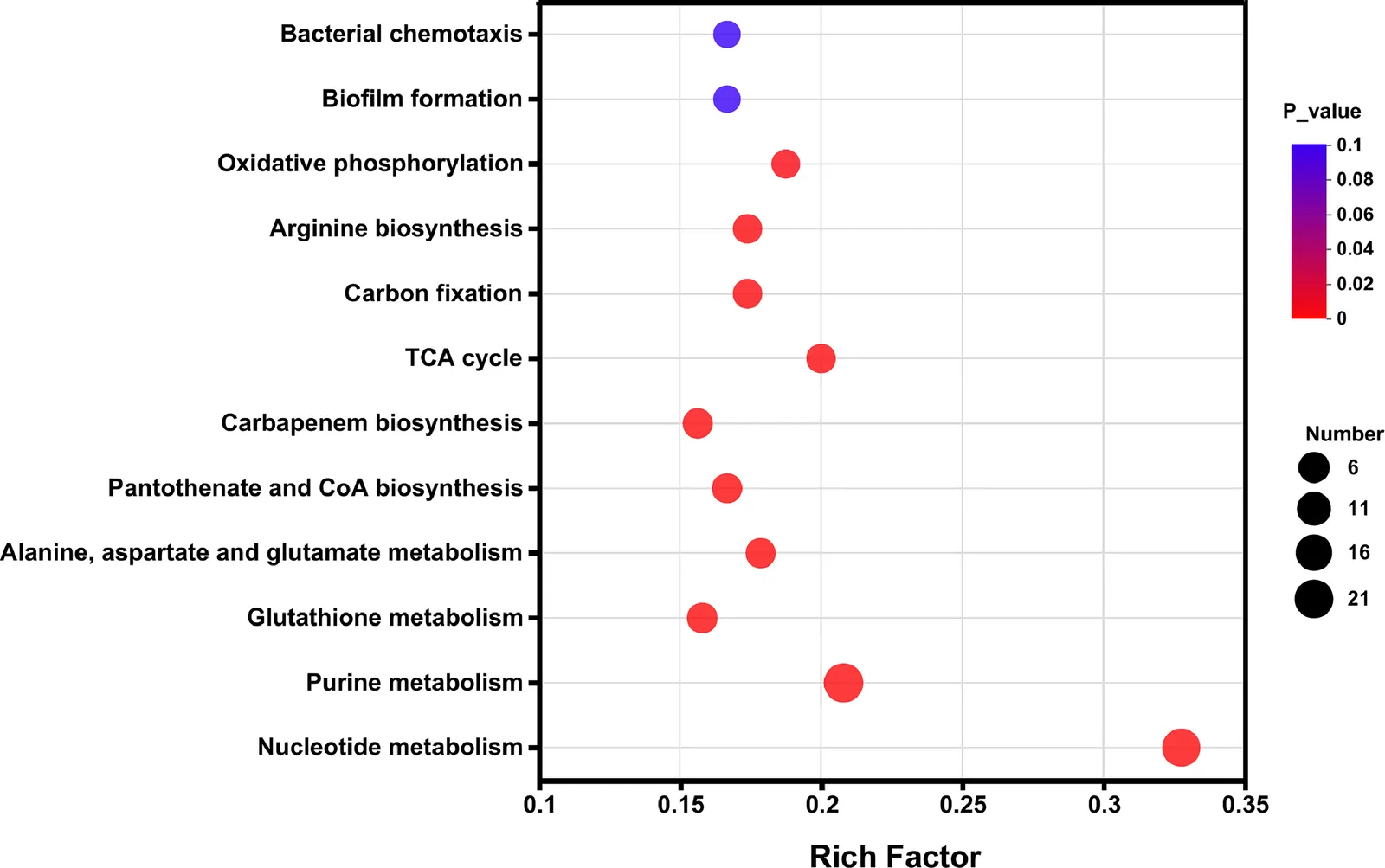
**The affected metabolic pathways in *E. coli* treated with BD/Vis.** The size of the bubble shows the number of differential metabolites. The color of the bubble shows the significance level.

The metabolism analysis indicates that *E. coli* experienced oxidative stress when treated with BD/Vis, as evidenced by: (1) Upregulation of glutathione metabolites like γ-glutamylcysteine, cysteinylglycine, and oxidized glutathione (GSSG) (Fig. S11b online), indicating activation of the glutathione antioxidant defense against ROS [42]; (2) Disruption of central metabolic pathways like glycolysis and the TCA cycle, consistent with the bacterial response to oxidative damage [43]. Changes in GSH and ATP concentrations further confirmed those findings (Fig. S12 online).

Additionally, the results suggest DNA damage occurred: (1) Reduction in nucleic acid metabolites like GMP, UMP, and AMP indicates inhibited RNA synthesis, which affects gene transcription and translation; (2) Elevated levels of nucleic acid bases (guanine, adenine) and nucleosides (deoxyadenosine, adenosine, guanosine) suggests nucleic acid repair was activated in response to damage [44].

In summary, multiple lines of evidence indicate BD/Vis treatment induced oxidative stress and subsequent DNA damage in *E. coli*. The bacterial response aimed to mitigate ROS accumulation but was ultimately insufficient to prevent nucleic acid destruction. For clarity, a network map was created to visualize the interconnections between these key metabolic pathways (Fig. 5). The bacterial disinfection mechanism of the BD/Solar system can then be summarized as follows (Fig. 6): Acetyl peroxyl radicals, produced by BD under solar irradiation, attack *E. coli*, propelling the bacteria into a state of oxidative stress characterized by raised intracellular ROS levels, which may be the main driving force for inactivation. To counteract this oxidative stress, the bacteria upregulate defense mechanisms, including the antioxidant protein SOD and the glutathione system. ROS compromised the bacterial cell membrane, leading to the production of oxidative damage markers such as MDA. Nucleic acid is also oxidatively damaged during the disinfection process. As the disinfection process proceeded, the accumulation of ROS surpassed the capacity of the antioxidant system. Consequently, *E. coli* fails to sustain redox homeostasis, culminating in cell rupture and death. Finally, cellular contents, including ions (such as K^+^) and biological macromolecules (such as nucleic acids and proteins) are released into the surrounding medium.

**Fig. 5:**
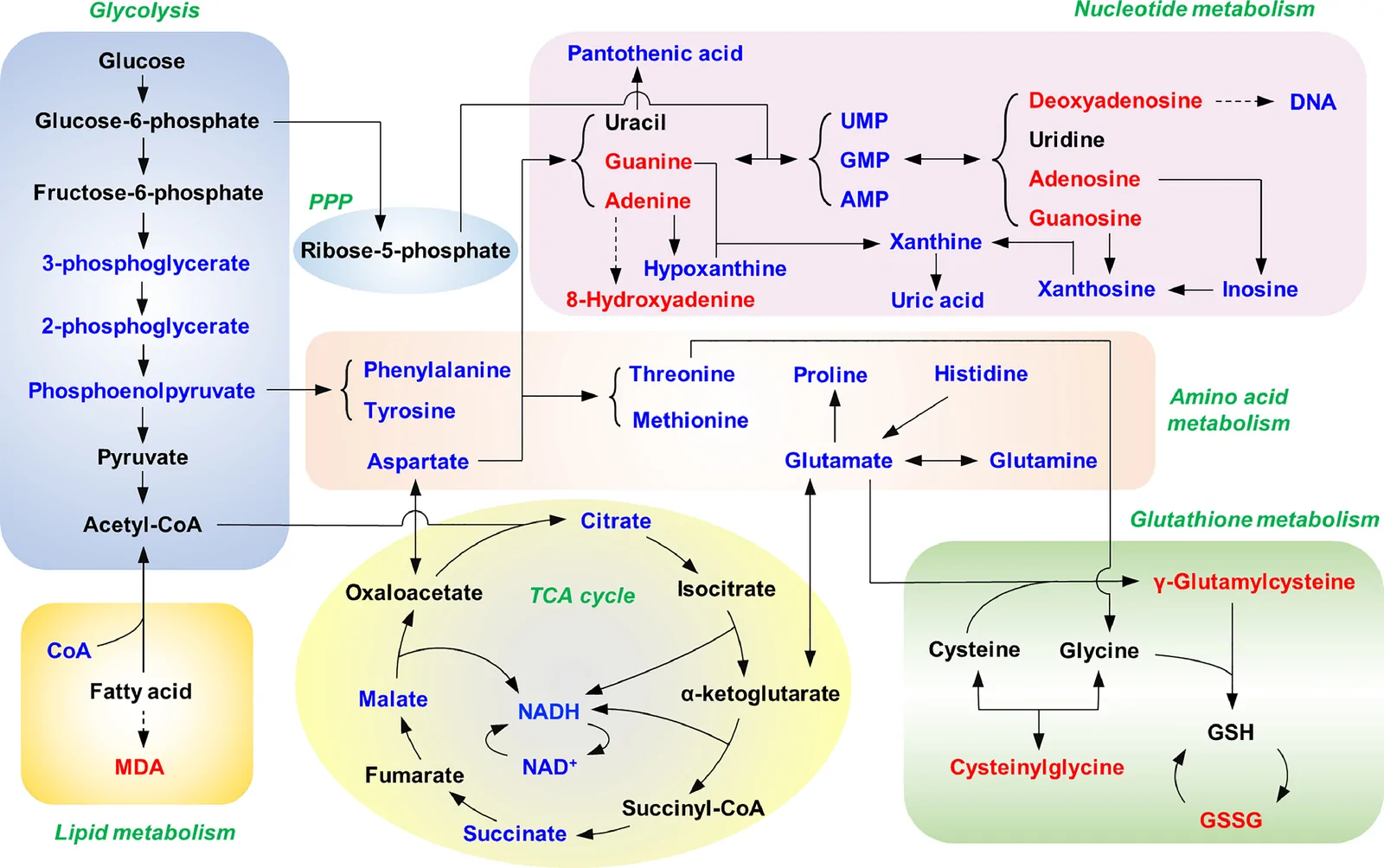
**Effects of BD/Vis on the main metabolic pathways in *E. coli* cells.** Font in black: not affected, font in red: up-regulated, font in blue: down-regulated. Solid arrows: direct transformation between metabolites, dashed arrows: putative metabolic processes. Abbreviations: NADH, nicotinamide adenine dinucleotide; GSH, glutathione; GSSG, oxidized glutathione; CoA, coenzyme A; MDA, malondialdehyde; UMP, uridine monophosphate; GMP, guanosine monophosphate; AMP, Adenosine monophosphate; PPP, pentose phosphate pathway.

**Fig. 6:**
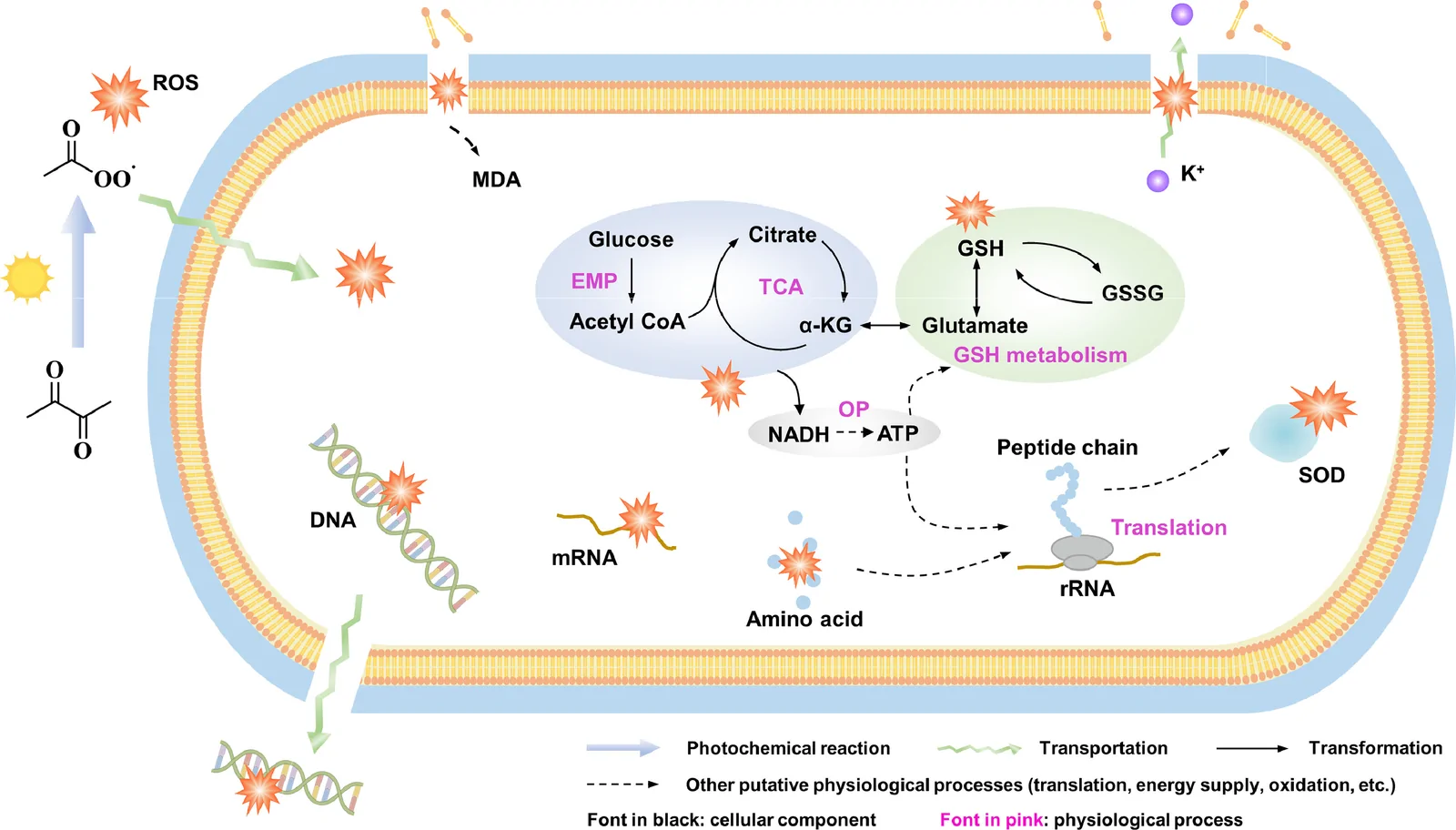
**Schematic illustration of the disinfection process in the BD/Solar system.** Abbreviations: EMP, glycolysis; α-KG, α-ketoglutarate; OP, oxidative phosphorylation.

### 3.5 Risks of water after disinfection treatment

Disinfection is an essential prerequisite for ensuring the microbiological safety of water; however, the application of disinfectants can lead to the formation of harmful DBPs. BD has been shown to have low toxicity [[17], [18], [19], [20]], and its photolysis products, consisting mainly of small molecular acids and alcohols [24], contribute to the overall safety of this disinfectant. A certain concentration of residual chlorine is present in tap water from the municipal network to ensure the microbiological safety of the water supply, which results in the presence of a certain concentration of DBPs. In the absence of any added disinfectant, seven DBPs were identified (Fig. 7 and Table S6 online), likely due to the residual chlorine in the tap water. After solar irradiation, the concentrations of all DBPs decreased, probably due to photolysis of DBPs [28]. When NaClO (3 mg/L Cl_2_) was added to the water, there was an observed increase in the concentration of all seven DBPs by 1.5 to 3.7 times (the difference in the DBP levels was due to the difference in the formation potential and the volatility of different types of DBPs), along with the detection of two DBPs (1,1,1-trichloropropanone (TCP) and bromodichloroacetic acid (BDCAA)). After about 3 h, the chlorine decayed rapidly to below 1 mg/L, which is the minimum limit for chlorine concentration in swimming pools (Fig. S13a online) [45]. This necessitates regular replenishment to maintain effective disinfection, which could lead to higher DBP levels. Conversely, the introduction of BD up to 0.5 mM did not significantly change the concentration of detected DBPs. On the other hand, acetyl peroxyl radicals did not seem to contribute to the degradation of DBPs, probably limited by the low concentration of the acetyl peroxyl radicals and the relatively stable structure of DBPs. Moreover, the depletion of BD was much slower, with significant amounts remaining after an 8-hour reaction period (Fig. S13b online), which will exert continuous microbial control. The slightly decreased pH during the BD/Solar disinfection process (Text S7 online) may increase the formation of DBPs [46], but this was not expected to have a negative impact on the water treatment process, given the buffering effect of natural water. To some extent, BD/Solar is similar to PAA disinfection in two aspects: (1) no additional formation of aliphatic DBPs [6,40], and (2) introduction of organic carbon to water. Further non-target analysis of other potentially harmful by-products is warranted. In addition, when it comes to human exposure (e.g., swimming pools), the potential health effects of acetyl peroxyl radicals under environmental exposure conditions need to be investigated further.

**Fig. 7:**
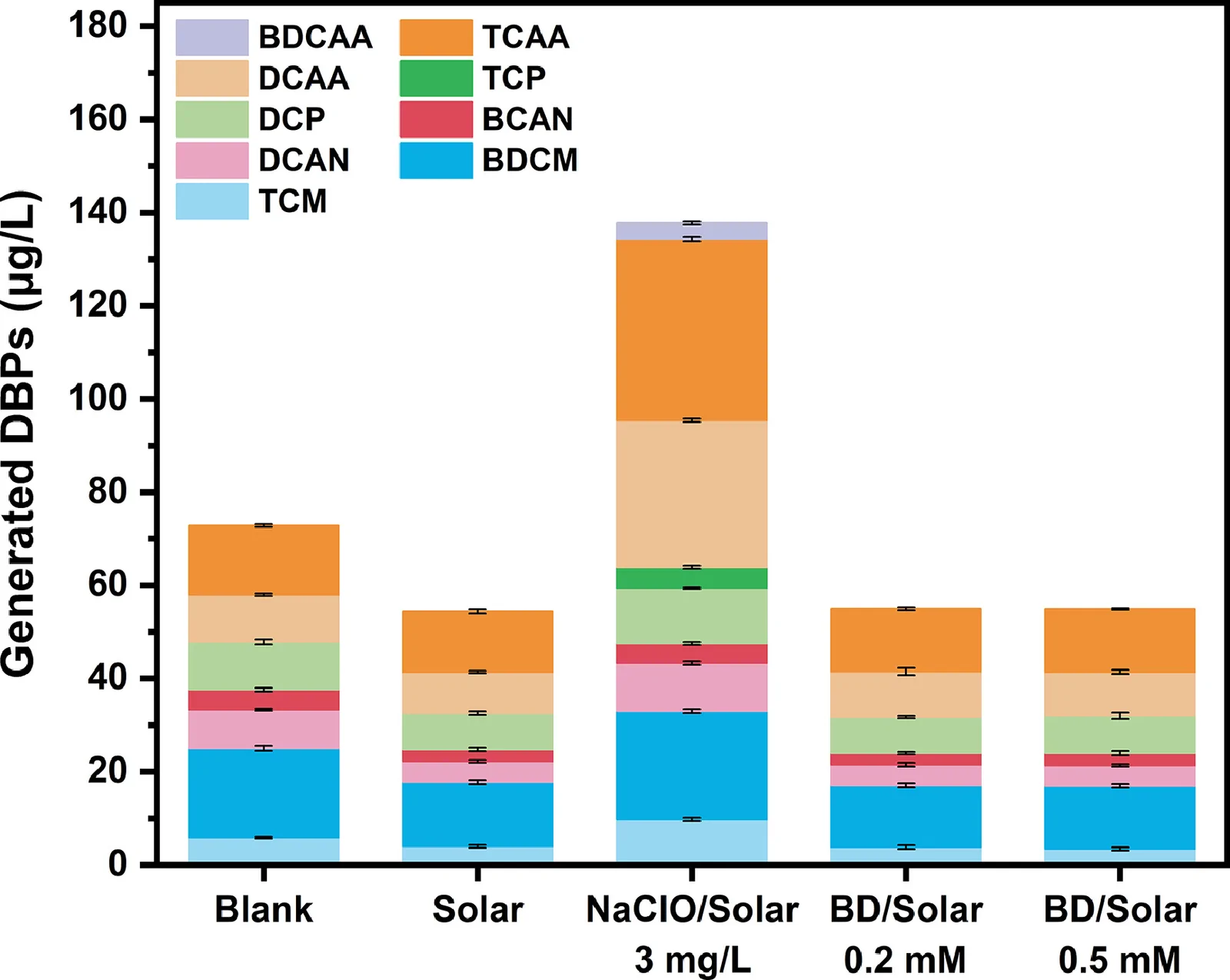
**Generated DBPs in simulated swimming pool water under solar irradiation for 8h.** Blank refers to the raw simulated swimming pool water without any treatment. Abbreviations: BDCAA, bromodichloroacetic acid; TCAA, trichloroacetic acid; DCAA, dichloroacetic acid; TCP, 1,1,1-trichloropropanone; DCP, 1,1-dichloropropanone; BCAN, bromochloroacetonitrile; DCAN, dichloroacetonitrile; BDCM, bromodichloromethane; TCM, trichloromethane.

## 4 Conclusion

BD, a food-use additive, could serve as a safe and energy-efficient water disinfectant when activated by sunlight. At equivalent concentrations (0.2 mM), the inactivation performance of BD surpassed conventional disinfectants like H_2_O_2_, PDS, and PI. Significantly, the addition of BD for water disinfection under solar irradiation does not increase the formation of halogenated DBPs in simulated swimming pool water. The BD/Solar process works by generating acetyl peroxyl radicals that can damage cell membranes, increase intracellular reactive oxygen species, disrupt cellular defenses, cause oxidative damage to nucleic acids, and ultimately rupture the cell membrane. Except for the potential for swimming pool disinfection, further research is necessary to assess the full potential of BD/Solar for practical applications, including its applicability to complex microbial communities, long-term effects, optimal operation conditions (distribution, stability, light penetration, etc.), and the way for integration with existing water treatment systems.

## CRediT authorship contribution statement

**Zewen Ji:** Data curation, Formal analysis, Investigation, Methodology, Visualization, and Writing—original draft; **Wenchang Zhang:** Data curation, Methodology; **Jianghua Yang:** Formal analysis, Methodology; **Peng Peng:** Investigation; **Haoran Chen:** Data curation, Methodology; **Shujuan Zhang:** Conceptualization, Supervision, Funding acquisition, Resources, Validation, Writing—review and editing.

## Declaration of competing interest

All the authors declare that they have no conflicts of interest in this work.

## Appendix Supplementary materials

Image, application 1
