# Supplementary material for: Solar-driven butanedione-based disinfection: A by-product-free approach to pathogen control
Source: Fundam Res. 2025 Mar 14;6(5):2950–8. doi: 10.1016/j.fmre.2025.03.003 (PMC13644404; doi:10.1016/j.fmre.2025.03.003)
Supplement: Supplementary file 1 [file mmc1.docx]

Supplementary materials for

**Solar-driven butanedione-based disinfection: A by-product-free approach to pathogen control**

Zewen Ji, Wenchang Zhang, Jianghua Yang, Peng Peng, Haoran Chen, Shujuan Zhang*

State Key Laboratory of Pollution Control and Resource Reuse, School of the Environment, Nanjing University, Nanjing 210023, China

*Correspondence author. Phone: +86 25 8960389, E-mail: [sjzhang@nju.edu.cn](mailto:sjzhang@nju.edu.cn)

Submitted to: ***Fundamental Research***

**This file includes:**

(1) Texts S1 through S8;

(2) Tables S1 through S6;

(3) Fig. S1 through S13;

(4) References

**Text S1. Chemicals**

BD, hippuric acid, uric acid, and citric acid were purchased from Macklin Biochemical Co. Ltd. (Shanghai, China). PI, PDS, H_2_O_2_, NaCl, NaHCO_3_, CaCl_2_, MgCl_2_, CH_3_COONa, Na_2_SO_4_, NaNO_3_, NaOH, HCl, D_2_O, and NaClO were purchased from Sinopharm Co. Ltd. (Shanghai, China). Suwannee River DOM (SR, Product No.1R101N) was obtained from the International Humic Substances Society (IHSS). Creatinine, and tert-butyl alcohol (TBA) were purchased from Aladdin Biochemical Technology Co. Ltd. (Shanghai, China). 2,4-hexadiene (2,4-HD) was purchased from Tokyo Chemical Industry Co. Ltd. (Japan). 5,5-dimethyl-1-pyrrolidine-N-oxide (DMPO) was obtained from Dojindo Co. Ltd. (Japan). Four THMs (trichloromethane (TCM), dichloroiodinemethane (DCIM), tribromomethane (TBM), bromodichloromethane (BDCM)) were purchased from ROE Scientific (USA). Five HAAs (bromodichloroacetic acid (BDCAA), dichloroacetic acid (DCAA), and trichloroacetic acid (TCAA), dibromoacetic acid (DBAA), tribromoacetic acid (TBAA)) were purchased from Sigma-Aldrich (USA). Four HANs (bromochloroacetonitrile (BCAN), dichloroacetonitrile (DCAN), monobromoacetonitrile (MBAN), tribromoacetonitrile (TBAN)) were purchased from J&K Scientific Ltd. (Shanghai, China). 1,1-dichloropropanone (DCP) and 1,1,1-trichloropropanone (TCP) were purchased from Supelco (USA). Methyl t-butyl ether (MTBE) and acetonitrile were liquid chromatography grade and purchased from Merck (Germany). High-purity oxygen and nitrogen (O_2_, N_2_) were purchased from Tianze Gas Co. Ltd. (Nanjing, China). These reagents were used without further purification. Ultrapure water (18.25 MΩ·cm) was produced by a water purification system (Shanghai Ulupure Industrial Co. Ltd., China).

**Text S2. Disinfection experiments**

In a typical bacterial inhibition experiment, bacterial suspension with an initial bacterial density of 1.0 × 10^7^ CFU/mL was added to a quartz reaction tube. A multi-channel photocatalytic reaction system (PCX-50C, PerfectLight, China) was used for the bacterial inhibition experiments. An LED light was used as the visible light source. The emission spectra of both the Vis-LED and natural sunlight (Solar) were recorded using a fiber optic spectrometer (AULTT-P4000, CEAULight, China).

Light intensity will affect the performance of photo-disinfection systems. The ratio of the number of molecules undergoing photochemical conversion per unit time to the total number of photons absorbed by the system is a constant value, also known as photonic efficiency [1]. According to the Lambert-Beer law, for a stationary solution, the absorbed photon irradiance (*I*_a_) is proportional to the initial photon irradiance (*I*_0_) at the given wavelength at the surface of the sample. In other words, the light intensity would affect the photoactivation of BD in a proportional way. However, it should be noted that the waters generally contain complicated matrix. The inner filtration effect varies with altered light intensity. Therefore, the apparent disinfection performance might not be proportionally changed with the light intensity.

Except for the light source, the influences of several other factors, including the BD dosage, the initial cell density, and the water matrix, on the performance of BD/Solar were also investigated with the univariate method. To assess the influence of BD dosage on bacterial inactivation, the experiment was conducted at three BD dosages: 0.1, 0.2, and 0.3 mM. To assess the impact of bacterial density on disinfection efficacy, the disinfection experiments were conducted at initial *E. coli* cell densities of 1 × 10^8^ and 1 × 10^6^ CFU/mL.

The humic acid (HA) used in the experiment was derived from the Suwannee River, which is often used to be a model for dissolved organic matter (DOM) in water. To prepare the solution, a certain amount of HA was dissolved in 0.1 mM sodium hydroxide solution and placed on a magnetic stirrer for 12 h. The solution was then filtered through a glass fiber membrane with a pore size of 0.45 μm. Concentrations reported were in terms of total organic carbon (TOC).

Photo-disinfection experiments were also conducted in natural waters, including tap water, Jiuxiang River water, and Tianlai Lake water (collected from Nanjing, China). All experiments were repeated at least three times.

Most of the experiments were based on the BD/Vis system. In addition, H_2_O_2_, PDS, and PI were used to make comparisons with the disinfection performance of BD under the same light and concentration (0.2 mM) conditions. Control experiments were conducted under BD alone (no light) or light alone (no BD added) conditions.

It should be noted that the photochemical reactions were determined by the photon absorbed by the reagents. Regardless of which light source was used, BD can absorb photons of wavelength up to 460 nm (**Fig. S1**). There was an overlap between the Vis and Solar spectra in this area (the shaded area in **Fig. S1**). Therefore, the conclusions drawn from the BD/Vis system can be generalized to the BD/Solar system.

**Text S3. Determination of disinfection kinetics**

The disinfection kinetics was fitted with the Hom model as **Eq. S1**:

$\log(N/N_{0})=-k\cdot c\cdot t^{n}=-k_{\text{obs}}\cdot t^{2}$ (S1)

where *N* is the cell density (CFU/mL), *k* is the disinfection rate constant (M^-1^ min^-2^), *c* is the disinfectant concentration (M), and *t* corresponds to the disinfection time (min). The *k*_obs_ is the observed rate constant (min^-2^).

As illustrated in **Fig. 1c**, the disinfection kinetics were slow at the initial 15 min due to the presence of the defense system in bacteria, resulting in a shoulder-shaped disinfection curve. However, faster disinfection kinetics were observed after 15 min. The parameter *n* is an empirical constant. Typically, a tailing-off effect is produced at the end of the disinfection process when *n* is less than 1 [2]. For *n* equals to 1, the Hom model simplifies to the Chick-Watson linear equation relating time to log(*N*/*N*_0_), which contrasts with the initially slow kinetics of the disinfection process [3]. When the *n* of the Hom model is greater than 1, the inactivation kinetic curve displays an initial shoulder which can more accurately describe the disinfection process [4]. To validate this, *n* values of 0.5, 1, 2, and 3 were used in the Hom model to analyze disinfection kinetics in the BD/Vis system (**Fig. S2**). Among these scenarios, the Hom model with *n* equal to 2 had the highest *R*^2^ value (*R*^2^ = 0.99), providing a better theoretical fit for the shoulder at the beginning of the disinfection process. Therefore, *n* was set to 2 in the Hom model for this research. In the experimental results, only *k*_obs_ was reported, which is the observed rate constant incorporating the disinfectant concentration *c*.

Due to the limitations of the plate counting method, it is not accurate to count the cell density below 10 CFU/mL (LOD). However, the number of colonies can still be counted in this situation. To better demonstrate the disinfection effect, the absence of colony formation was denoted as 1 (log1 = 0), to express the completion of the disinfection process. In addition, the points below LOD were excluded when fitting to the Hom model for *k*_obs_.

**Text S4.** **Analytic methods**

The samples for ESR analysis were prepared by mixing 10 mM BD with 100 mM DMPO in an oxygen-free chamber. The mixed solutions were exposed in situ to a Vis lamp at a light intensity of 50 mW/cm^2^. The ESR signals were recorded after 5 min irradiation. The specific parameters used for ESR analysis were as follows: a center field of 3500 G, a sweep width of 100.0 G, a microwave frequency of 9.826 GHz, and a power output of 20 mW.

ROS assay kit (S0033, Boyotime) was used to analyze the changes in intracellular ROS during the disinfection process. 2’,7’-dichlorofluorescin diacetate (DCFH-DA) was used as a fluorescent probe to determine the intracellular ROS levels. The fluorescence intensity was measured at excitation and emission wavelengths of 488 nm and 525 nm using a microplate reader.

After ultrasonic fragmentation of the bacteria in the reaction suspension, superoxide dismutase (SOD) activity and malondialdehyde (MDA) content were assessed using assay kits (A001-3 and A003-1, Jiancheng). SOD activity can be detected by measuring the formation of a yellow WST-8 formazan dye, which absorbs light at a wavelength of 450 nm. This dye is produced through the reduction of •O_2_^–^, which is generated when xanthine and its corresponding oxidase are added. By quantifying the percentage of inhibition in the formation of this dye, the level of SOD activity can be determined. MDA condenses with thiobarbituric acid (TBA) under acidic and high-temperature conditions to form a brownish-red adduct with a maximum absorption wavelength of 532 nm. Absorbance measurements can be performed to estimate the amount of MDA in a sample.

After the extraction of bacterial DNA using the Bacterial Genomic DNA Extraction Kit (DM1600, Solarbio), the oxidative damage of genomic DNA during disinfection was further analyzed using the 8-hydroxydeoxyguanosine (8-OHdG) ELISA Kit (SEKSM-0038, Solarbio). Standard samples and specimens were added to an enzyme-labeled well pre-coated with 8-OHdG antigen. After incubation, biotin-labeled anti-8-OHdG antibody was added. Then, it was combined with HRP-labeled streptavidin to form an immune complex. After further incubation and washing, unbound enzymes were removed. Subsequently, a colorimetric substrate TMB was added, which turned blue and eventually converted to yellow under acidic conditions. Finally, the absorbance (OD) value of the reaction well sample was measured at 450 nm. The concentration of 8-OHdG in the sample was inversely proportional to the OD value. The concentration of 8-OHdG in the sample was calculated by constructing a standard curve.

The gene encoding *β*-galactosidase (*lacZ*) was amplified by a polymerase chain reaction (PCR) system to determine the concentration of nucleic acids released during the disinfection process. During the disinfection reaction, 30 mL of the reaction suspension was taken at certain intervals, and centrifuged at 8000 rpm for 10 min, and the supernatant and precipitate were collected separately. The supernatant was purified using a DNA Purification Kit (19106ES50, Yeasen) to obtain extracellular *lacZ* (e-*lacZ*). For extracting intracellular *lacZ* (i-*lacZ*), the Bacterial Genomic DNA Extraction Kit (DM1600, Solarbio) was used, following the same pre-treatment process as for 8-OHdG. PCR assays were conducted in a 9902 PCR System (Applied Biosystems, Thermo Fisher, USA). The specific primers for *lacZ* were provided by Sangon Biotech Corp., China. Specifically, the primer sets (5’-CGCATTATCCGAACCATCCGCTGTG-3’) were used as the forward primer and (5’-CAACGCTTATTACCCAGCTCGATGC-3’) were used as the reverse primer. The PCR amplification was conducted in a total volume of 20 μL containing 10 μL of Taq Master Mix, 1 μL of forward primers (20 µM), 1 μL of reverse primers (20 µM), 1 μL of DNA sample, and 7 μL of ddH_2_O. The PCR thermal cycle profile of the *lacZ* gene was as follows: 1 cycle at 94℃ for 10 min, 25 cycles at 94℃ for 30 s, 58℃ for 30 s, and 72℃ for 45 s, and 1 cycle at 72℃ for 10 min. The PCR products were verified by DNA agarose gel electrophoresis (0.6% agarose gel at 100 V for 20 min in 1×TAE buffer).

The K^+^ concentration in solution was determined using inductively coupled plasma emission spectrometry (ICP, Thermo Scientific).

The metabolite changes in *E. coli* during the inactivation treatment were determined by metabolomics. Metabolite extraction and analysis were performed by Majorbio Co. Ltd. (Shanghai, China). Statistical analysis of the results was performed on the Majorbio Cloud Platform (www.majorbio.com) [5]. Each set of experiments was repeated six times for biological replicates.

Glutathione (GSH) and adenosine triphosphate (ATP) concentrations were assessed using assay kits (S0053 and S0026, Boyotime). GSSG is reduced to GSH by glutathione reductase, and GSH reacts with the color-generating substrate DTNB to produce yellow TNB and GSSG. The amount of total glutathione (GSSG + GSH) determines the amount of yellow TNB formed, and thus the amount of total glutathione can be calculated by measuring the absorbance at 412 nm. The amount of GSH can be calculated by first removing GSH from the sample with an appropriate reagent and subtracting the amount of GSSG from the amount of total glutathione (GSSG + GSH). ATP Assay Kit is developed based on the fact that ATP is required to provide energy when firefly luciferase catalyzes the production of fluorescent light from fluorescein. When both firefly luciferase and fluorescein are in excess, the production of fluorescence is proportional to the concentration of ATP within a certain concentration range. The concentration of ATP in the sample was calculated from the standard curve.

**Text S5.** **Metabolite extraction and identification**

After treating *E. coli* of an initial concentration of 1 × 10^8^ CFU/mL in the BD/Vis system for 30 min, a 30-mL reaction suspension was centrifuged at 8000 rpm for 10 min, and the precipitate was collected and stored in a -80°C refrigerator.

For metabolite extraction, a 2 mL centrifuge tube was utilized. To this tube, a 6 mm diameter grinding bead and 50 mg of solid sample were added. The extraction solution consisted of methanol and water in a 4:1 (v:v) ratio, supplemented with an internal standard (L-2-chlorophenylalanine) at a concentration of 0.02 mg/mL. The samples were then subjected to grinding using the Wonbio-96c frozen tissue grinder (Shanghai Wanbo Biotechnology Co., LTD) for 6 minutes at -10°C and 50 Hz. Subsequently, low-temperature ultrasonic extraction was performed for 30 minutes at 5°C and 40 kHz. After extraction, the samples were kept at -20°C for 30 minutes, followed by centrifugation at 4°C and 13000 g for 15 minutes. The resulting supernatant was then transferred to an injection vial for LC-MS/MS analysis.

The sample was subjected to LC-MS/MS analysis at Majorbio Bio-Pharm Technology Co. Ltd. (Shanghai, China) using a Thermo UHPLC-Q Exactive HF-X system equipped with an ACQUITY HSS T3 column from Waters, USA (100 mm × 2.1 mm i.d., 1.8 μm). The mobile phases consisted of solvent A, which was composed of 0.1% formic acid in water: acetonitrile mixture (95:5, v/v), and solvent B, which contained 0.1% formic acid in a mixture of acetonitrile: isopropanol: water (47.5:47.5:5, v/v). The flow rate was set to 0.40 mL/min, and the column temperature was maintained at 40℃.

Mass spectrometric data were acquired with a Thermo UHPLC-Q Exactive HF-X Mass Spectrometer equipped with an electrospray ionization (ESI) source operating in both positive and negative modes. Optimal conditions included a source temperature of 425℃, sheath gas flow rate at 50 arb, Aux gas flow rate at 13 arb, and ion-spray voltage floating (ISVF) set to -3500 V in negative mode and 3500 V in positive mode. The normalized collision energy ranged from 20-40-60 V during MS/MS analysis. The instrument had a full MS resolution of 60000 and an MS/MS resolution of 7500. Data acquisition utilized the Data Dependent Acquisition (DDA) mode, while detection spanned a mass range of 70–1050 m/z.

The raw data of LC-MS were imported into the metabolomics processing software Progenesis QI (Waters Corporation, Milford, USA) for preprocessing. The preprocessed information was matched with the metabolic public databases HMDB (http://www.hmdb.ca/) and Metlin (https://metlin.scripps.edu/) databases to obtain metabolite information.

Principal component analysis (PCA) was performed using ropls in *R* language (Version 1.6.2). The metabolites with variable importance in the projection (VIP) of > 1, *p*-value < 0.05, and a fold change (FC) of ≥ 1.2 or ≤ 0.8 were considered to be differential metabolites. Metabolic pathway enrichment was carried out by the Python package scipy.stats. The KEGG database was used to investigate the roles of various metabolites and metabolic pathways. The Rich Factor is the ratio of the number of differential metabolites under a metabolic pathway to the total number of metabolites annotated to that pathway. The metabolic pathways were considered statistically significant enrichment when the *p*-value of the metabolic pathway was < 0.05.

**Text S6. Analysis of DBPs**

Mainly 4 types of DBPs were measured, including four THMs (TCM, BDCM, DCIM, and TBM), four HANs (BCAN, DCAN, MBAN, and TBAN), two HKs (DCP and TCP), and five HAAs (BDCAA, DCAA, TCAA, DBAA, and TBAA). The former three types (THMs, HANs, HKs) of DBPs were extracted according to the EPA Method 551.1 [6]. The five HAAs were extracted according to the EPA Method 552.3 [6]. The sample preparation procedures were exactly the same as those in Reference [6]. All the samples were analyzed with GC-ECD. The details for GC-ECD analysis: A gas chromatography system (Agilent 7890A) equipped with a DB-1 capillary column (30 m × 0.25 mm × 1 μm film thickness) and an electron capture detector was used. The pretreated samples (1 μL) were injected in splitless mode. The injector and detector temperatures were 220°C and 320°C, respectively. The carrier gas (helium) and make-up gas (nitrogen) were at flow rates of 1.0 mL/min and 60 mL/min, respectively. The oven temperature program: initial temperature 35°C for 0 min; first ramp of 2 min to 140°C (6°C/min); and second ramp of 2 min to 230°C (20°C/min).

To determine the level of quantification (LOQ), we firstly determined the method detection level (MDL) as per the APHA Standard Methods for the Examination of Water and Wastewater. In brief, standard solutions of DBPs with concentrations close to the estimated MDLs were prepared and analyzed for seven portions over a period of 3 days. The standard deviation (SD) of the 7 replicates was calculated. It is important to note that the MDLs for different DBPs vary due to differences in the signal response of the instruments. Therefore, we assessed the SD across a range of concentrations from 0.1 to 1 μg/L. The MDL was then determined as 3.14 times the SD (3.14 is the one-sided student *t* value for 6 degrees of freedom (corresponding to 7 replicates) at the 99% confidence level). The calculated values closest to the estimated MDLs were taken as the analyte MDLs. Based on the relationship among the levels, the LOQs (typically the concentration that produces a signal of 10SD) of the tested DBPs were calculated as 3 times the MDLs (**Table S3**).

The calibration curves for DBPs were established in the concentration range of 0.1-50 μg/L with several data points in the low concentration range near LOQs. The calibration curves for all the tested DBPs are shown in **Fig. S3** and the corresponding linear fitting equations are listed in **Table S4**. For each tested DBP, the quantification reliability was also checked with recovery test. The recoveries (each for 3 replicates) for the majority of the DBPs ranged between 80% and 110% at 1 and 20 μg/L (**Table S3**).

**Text S7. Influencing factors in bacterial inactivation by BD/Solar**

BD dosage and initial cell density are two intrinsic factors in the BD/Solar disinfection process. When 0.1 mM of BD was used, no survival *E. coli* was observed within 60 min (*k*_obs_ = (1.9 ± 0.1) × 10^-3^ min^-2^) under Vis irradiation (**Fig. S4a** and **S4b**). As the BD dosage was increased to 0.2 and 0.3 mM, the required irradiation time was shortened to 45 and 30 min, with *k*_obs_ values increased to (3.4 ± 0.1) × 10^-3^ and (7.6 ± 0.2) × 10^-3^ min^-2^, respectively (**Fig. S4a** and **S4b**). At an initial BD dosage of 0.2 mM, only 14.2% BD was consumed after 60 min irradiation (**Fig. S4c**), indicating that the dosed BD is sufficient. Therefore, BD of 0.2 mM was taken for further studies to achieve a balance between efficiency and consumption.

To assess the impact of bacterial density on disinfection efficacy, the disinfection experiments were conducted at initial *E. coli* cell densities of 1 × 10^8^ and 1 × 10^6^ CFU/mL. The irradiation time required for a complete inactivation thus varied from 75 to 30 min with *k*_obs_ values of (6.5 ± 0.2) × 10^-3^ and (1.4 ± 0.1) × 10^-3^ min^-2^, respectively (**Fig. S5a** and **S5b**).

Bacterial cells, as well as the photolysis of BD, are sensitive to pH. The inactivation of *E. coli* by BD/Vis was indeed pH-dependent **(Fig. S5c)**. The process was more effective at low pH with *k*_obs_ values increased from (1.2 ± 0.1) × 10^-3^ min^-2^ (pH 9) to (30.8 ± 0.7) × 10^-3^ min^-2^ (pH 3) **(Fig. S5d)**. In addition, there was a slight decrease in solution pH after disinfection (from pH 7 to pH 5.6 and from pH 9 to pH 6). This is because the photolysis products of BD were mainly low molecular weight organic acids such as acetic acid [7].

DOM, such as HA, is widely present in natural water. While low concentrations of HA (1 mg/L) had a negligible effect on the disinfection efficacy, the presence of HA at higher concentrations (10 mg/L) deteriorated the inactivation performance (the value of *k_obs_* was decreased from (3.4 ± 0.1) × 10^-3^ min^-2^ to (1.04 ± 0.04) × 10^-3^ min^-2^) **(Fig. S6a** and **S6b)** due to its competition with BD for light absorption [8]. Nevertheless, a 3.42 log reduction was still achieved within 60 min in the presence of 10 mg/L HA.

Among the common ions in water, Cl^-^ and NO_3_^-^ exerted negligible effects on *E. coli* inactivation by BD/Vis, whereas SO_4_^2–^, CH_3_COO^–^, Mg^2+^, and Ca^2+^ inhibited the inactivation to some extent (**Fig. S6c** and **S6d**). The effect of HCO_3_^–^ was the most prominent (*k*_obs_ = (1.22 ± 0.05) × 10^-3^ min^-2^). This is because HCO_3_^-^ can react with ROS [9], resulting in the depletion of the ROS and thus inhibiting the disinfection. Even so, the majority of *E. coli* (4.43 log) was still inactivated at 60 min.

Compared with ultrapure water, the irradiation time taken to completely inactivate *E. coli* in synthetic swimming pool water, Jiuxiang River, and Tianlai Lake were increased to 60, 75, and 90 min with the *k*_obs_ values decreased to (1.9 ± 0.1) × 10^-3^, (1.6 ± 0.1) × 10^-3^, and (0.86 ± 0.05) × 10^-3^ min^-2^, respectively (**Fig. S6e** and **S6f**). The inhibitory effects might come from either the inner filtration effect (competition for photon absorption) or free radical competition reactions caused by the water matrix (**Table S1**) [10].

**Text S8. Metabolism analysis**

Within glutathione metabolism, we observed significant upregulation of γ-glutamylcysteine, cysteinylglycine, and oxidized glutathione (GSSG), indicating that *E. coli* was under oxidative stress. Glutathione (GSH) serves as a critical non-enzymatic antioxidant, scavenging oxygen radicals with its thiol groups to maintain redox balance and mitigate ROS accumulation [11,12]. This finding aligns with the observed increase in ROS and SOD activity (**Fig. 2h-i**), further supporting the activation of the glutathione antioxidant defense. The trend of GSH concentration was consistent with the above hypothesis. The GSH concentration increased slightly within 15 min and decreased gradually thereafter (**Fig. S12a**). These results further confirmed that the glutathione antioxidant defense system was activated in bacteria under oxidative stress. Meanwhile, GSH depletion promoted the accumulation of GSSG and inhibited the antioxidant capacity of bacteria. In addition, it should be noted that at 30 min, the GSH concentration decreased to a level slightly lower than the initial level, but did not meet the selection conditions for differential metabolites (FC > 0.8).

Glycolysis, the pathway by which glucose is metabolized to produce energy [13], was disrupted, as evidenced by the downregulation of intermediates like 3-phosphoglycerate, 2-phosphoglycerate, and phosphoenolpyruvate. The TCA cycle, a central energy-producing pathway [13], also showed downregulation in key intermediates such as citrate, malate, and succinate. Correspondingly, oxidative phosphorylation, which converts NADH into ATP [14], was compromised due to reduced levels of NADH and NAD^+^. The down-regulation of these metabolites was consistent with the observation that the concentration of ATP decreased progressively with the disinfection process (**Fig. S12b**). Furthermore, the down-regulation of CoA, a molecule associated with lipid metabolism, suggests a broad impairment of central carbon metabolism, potentially exacerbated by lipid peroxidation as indicated by increased MDA levels (**Fig. 2j**).

The observed decrease in energy metabolism is consistent with the bacterial response to oxidative stress [13,15], which may also impede other energy-demanding processes, such as the synthesis of antioxidant proteins. This could further intensify the oxidative stress experienced by the bacteria.

In addition to insufficient energy supply, the levels of amino acids, the raw materials for the synthesis of antioxidant enzymes, were also reduced, which further exacerbated the redox homeostasis imbalance (**Fig. 2i**) [16]. Notably, glutamate is involved in a wide range of bacterial metabolic processes, including responses to environmental stress, especially glutathione synthesis. Proline can act as a signaling molecule to protect cells by removing reactive oxygen species [17]. The depletion of glutamate and proline, both of which are implicated in stress responses and antioxidant defense, suggests a diminished capacity to counteract oxidative damage.

The metabolic pathways of nucleotide and purine metabolism were profoundly impacted by BD/Vis treatment. The observed decrease in metabolites such as xanthine, hypoxanthine, and uric acid, which are end products of purine degradation, alongside a reduction in pantothenic acid, a marker of pyrimidine catabolism, suggests an inhibition of base catabolism [16]. Nucleotides are the basic units that make up RNA, and the reduction of three nucleotides (GMP, UMP, and AMP) suggests that RNA synthesis is blocked, which in turn affects the transcription and translation processes of the cell.

Considering the result that the content of most amino acids was reduced, the hypothesis that the synthesis of antioxidant proteins in bacteria is hindered is further verified. At the same time guanine, adenine, deoxyadenosine, adenosine, and guanosine levels were elevated, and these results suggest that the nucleic acids of *E. coli* are damaged. They may maintain the repair process of nucleic acids (DNA and RNA) by decreasing base catabolism. In addition, a significant increase in the level of 8-hydroxyadenine, an oxidation product of adenine, was detected, which further suggests an oxidative attack on nucleic acids. Thus, from a metabolomics point of view, the BD/Vis treatment ultimately led to the destruction of intracellular nucleic acids.

**Table S1.** The water quality parameters of natural waters

| **Parameter** | **Units** | **Tap water** | **Lake water** | | **River water** |
| --- | --- | --- | --- | --- | --- |
| pH | -- | 7.88 | | 8.05 | 7.93 |
| DO | mg/L | 9.13 | | 9.38 | 9.59 |
| TOC | mg/L | 1.08 | | 5.72 | 2.23 |
| TN | mg/L | 0.11 | | 1.31 | 0.28 |
| Na^+^ | mg/L | 4.7 | | 29.7 | 16.6 |
| Cl^-^ | mg/L | 7.2 | | 33.1 | 24.6 |
| NO_3_^-^ | mg/L | 7.7 | | 22.3 | 13.9 |

**Table S2.** The formulation of artificial body fluids

| **Constituent** | **Concentration (mg/L)** |
| --- | --- |
| NH_4_Cl | 2000 |
| Urea | 14800 |
| L-Histidine | 1210 |
| Hippuric acid | 1710 |
| Uric acid | 490 |
| Citric acid | 640 |
| NaH_2_PO_4_ | 4300 |
| Creatinine | 1800 |

**Table S3.** Retention time (RT), level of quantification (LOQ), recovery, and relative standard deviation (RSD) in DBPs analysis

| **DBP** | **RT (min)** | **LOQ (µg/L)** | **Recovery (RSD, n=3)** | |
| --- | --- | --- | --- | --- |
|  |  |  | **1 μg/L*^a^*** | **20 μg/L** |
| TCM | 4.32 | 0.27 | 92.6% (4.2%) | 96.2% (3.4%) |
| DCAN | 5.95 | 0.33 | 109.3% (3.5%) | 97.4% (4.7%) |
| BDCM | 6.12 | 0.39 | 96.4% (5.2%) | 103.5% (3.9%) |
| DCP | 6.75 | 0.36 | 110.1% (3.1%) | 93.5% (2.9%) |
| MBAN | 7.15 | 0.45 | 103.6% (4.2%) | 95.3% (5.1%) |
| BCAN | 8.37 | 0.24 | 110.3% (1.5%) | 104.1% (3.6%) |
| DCIM | 9.31 | 0.57 | 102.4% (2.3%) | 106.3% (5.3%) |
| TCP | 10.07 | 0.33 | 81.9% (1.4%) | 85.2% (3.4%) |
| TBM | 11.15 | 0.54 | 78.6% (4.2%) | 82.7% (4.2%) |
| TBAN | 14.71 | 0.42 | 92.7% (6.1%) | 99.6% (3.7%) |
| DCAA | 9.78 | 0.81 | 107.1% (3.6%) | 109.3% (3.8%) |
| TCAA | 12.31 | 0.63 | 106.5% (5.8%) | 107.2% (1.2%) |
| DBAA | 14.43 | 0.51 | 111.3% (8.6%) | 106.2% (3.7%) |
| BDCAA | 14.87 | 0.66 | 93.2% (4.1%) | 88.7% (4.4%) |
| TBAA | 19.76 | 0.78 | 114.2% (1.3%) | 91.3% (2.9%) |

*^a^* Date sources: Reference [6].

**Table S4.** The calibration curves for DBPs in the concentration range of 0.1-50 μg/L

| **DBP** | **Calibration curve** *^a^* | ***R*^2^** |
| --- | --- | --- |
| TCM | *y* = 3114*x* + 181 | 0.9995 |
| DCAN | *y* = 1617*x* - 450 | 0.9988 |
| BDCM | *y* = 1625*x* + 60 | 0.9984 |
| DCP | *y* = 1617*x* + 251 | 0.9989 |
| MBAN | *y* = 1328*x* + 315 | 0.9992 |
| BCAN | *y* = 4878*x* - 594 | 0.9989 |
| DCIM | *y* = 819*x* + 346 | 0.9969 |
| TCP | *y* = 1939*x* - 262 | 0.9996 |
| TBM | *y* = 365*x* + 92 | 0.9991 |
| TBAN | *y* = 1879*x* + 483 | 0.9975 |
| DCAA | *y* = 260*x* - 54 | 0.9993 |
| TCAA | *y* = 647*x* + 103 | 0.9948 |
| DBAA | *y* = 918*x* - 212 | 0.9978 |
| BDCAA | *y* = 673*x* - 153 | 0.9949 |
| TBAA | *y* = 329*x* - 96 | 0.9955 |

*^a^* *y* represents Peak area and *x* is Concentration (μg/L).

**Table S5.** Disinfection efficiency (DE) and equivalent cost (EC) of variant disinfection methods

| **System** | **DE**  **(CFU/mL)** | **Dosage** | **Time**  **(min)** | **EC*^a^***  **(×10^-12^)** | **Ref.** |
| --- | --- | --- | --- | --- | --- |
| H_2_O_2_/UVA | 6-log | 10 mg/L H_2_O_2_ | 12 | 18.9 | 18 |
| H_2_O_2_/Fe^2+^ | 6-log | 0.6 mg/L FeSO_4_·7H_2_O  10 mg/L H_2_O_2_ | 90 | 142.2 | 19 |
| PDS/VL | 7-log | 546 mg/L PDS | 120 | 135.0 | 20 |
| PAA/UV | 7.7-log | 71 mg/L PAA | 5 | 6.3 | 3 |
| PI/NSL | 7-log | 42.8 mg/L PI | 15 | 9.5 | 21 |
| BD/Vis | 7-log | 17.2 mg/L BD | 45 | 4.3 | This work |
| BD/Solar | 7-log | 17.2 mg/L BD | 15 | 1.4 | This work |

*^a^* EC $= \frac{Time \times\Sigma\left( Dosage \times Price \right)}{10^\mathrm{DE}} (min USD/CFU)$	(S2)

Reagent prices: (Data from Aladdin^®^ and Adamas^®^)

| 30% H_2_O_2_ | BD | PDS | PI | 35% PAA | FeSO_4_**·**7H_2_O |
| --- | --- | --- | --- | --- | --- |
| $26.2  /500 mL | $27.3  /500 mL | $10.3  /500 g | $74.2  /500 g | $190.2  /500 mL | $5.8  /500 g |

Note that the cost of UV could hardly be evaluated and thus the cost was evaluated based on the price of chemicals.

**Table S6.** Concentration of DBPs in tested water samples (in units of μg/L)

| **DBP** | **Blank** | **Solar** | **NaClO/Solar**  **3 mg/L** | **BD/Solar**  **0.2 mM** | **BD/Solar**  **0.5 mM** |
| --- | --- | --- | --- | --- | --- |
| TCM | 5.9 ± 0.2 | 4.0 ± 0.3 | 9.8 ± 0.4 | 3.8 ± 0.4 | 3.4 ± 0.4 |
| BDCM | 19.2 ± 0.6 | 13.8 ± 0.5 | 23.2 ± 0.4 | 13.2 ± 0.5 | 13.5 ± 0.4 |
| DCAN | 8.3 ± 0.2 | 4.5 ± 0.3 | 10.3 ± 0.4 | 4.4 ± 0.4 | 4.4 ± 0.3 |
| BCAN | 4.3 ± 0.4 | 2.5 ± 0.4 | 4.2 ± 0.3 | 2.5 ± 0.3 | 2.6 ± 0.5 |
| DCP | 10.3 ± 0.6 | 7.8 ± 0.4 | 11.9 ± 0.2 | 7.7 ± 0.3 | 8.0 ± 0.7 |
| TCP | N.D.*^a^* | N.D. | 4.5 ± 0.4 | N.D. | N.D. |
| DCAA | 10.0 ± 0.3 | 8.8 ± 0.3 | 31.5 ± 0.4 | 9.8 ± 0.8 | 9.4 ± 0.4 |
| TCAA | 14.9 ± 0.3 | 13.0 ± 0.5 | 38.8 ± 0.5 | 13.4 ± 0.3 | 13.5 ± 0.2 |
| BDCAA | N.D. | N.D. | 3.5 ± 0.4 | N.D. | N.D. |

*^a^* Not detected.

**Fig. S1.** UV-vis absorption spectra of reagents (Left) and the emission spectra of the employed Vis-LED and Solar (Right).

**Fig. S2.** Linear correlation between -log(*N*/*N*_0_) and *t^n^* with *n* = 0.5 (a), 1 (b), 2 (c) and 3 (d).

**Fig. S3.** The calibration curves for DBPs in the concentration range of 0.1–50 μg/L.

**Fig. S4.** Effects of initial BD dosage on the disinfection performance of *E. coli* in the BD/Vis system. (a) Kinetic profile of bacterial inactivation, (b) The observed inactivation rate constant, (c) The consumption of BD during the disinfection process.

**Fig. S5.** Disinfection kinetic profile (a, c) and rate constant (b, d) of BD/Vis for *E. coli* at different initial cell densities and solution pHs.

**Fig. S6.** Disinfection kinetic profiles (a, c, e) and rate constants (b, d, f) of BD/Vis for *E. coli* inactivation at different conditions. Initial cell density: 1 × 10^7^ CFU/mL, [BD]_0_: 0.2 mM, inorganic salts: 15 mM (for c and d).

**Fig. S7.** The regrowth of *E. coli* cells after the disinfection process. Initial cell density: 1 × 10^7^ CFU/mL, [BD]_0_: 0.2 mM.

**Fig. S8.** The observed rate constant of *E. coli* disinfection under different aeration conditions (a) or in the presence of scavengers (b).

**Fig. S9.** Effects of scavengers on bacterial inactivation performance by Vis-only. [2,4-HD]: 10 mM, [TBA]: 10 mM, [SOD]: 50 U/mL

**Fig. S10.** PCA cluster analysis of the metabolites in BD/Vis-treated (30 min) and control group.

**Fig. S11.** Differential metabolites in *E. coli* cells treated with BD/Vis for 30 min. (a) Volcano plots. (b) Relative abundance of differential metabolites.

**Fig. S12.** Change of relative level of GSH (a) and ATP (b) during the disinfection process. Initial cell density: 1 × 10^8^ CFU/mL, [BD]_0_: 0.2 mM.

**Fig. S13.** Change in concentration of disinfectant: NaClO (a) and BD (b).

**References**

[1]	B. Wu, M. Yang, R. Yin, et al., Applicability of light sources and the inner filter effect in UV/acetylacetone and UV/H_2_O_2_ processes, J. Hazard. Mater. 335 (2017) 100-107.

[2]	F. Liu, Y. Hou, S. Wang, et al., Periodate activation by pyrite for the disinfection of antibiotic-resistant bacteria: Performance and mechanisms, Water Res. 230 (2023) 119508.

[3]	T. Zhang, T. Wang, B. Mejia-Tickner, et al., Inactivation of bacteria by peracetic acid combined with ultraviolet irradiation: Mechanism and optimization, Environ. Sci. Technol. 54 (2020) 9652-9661.

[4]	L. Cao, J. Wang, Z. Wang, et al., Inactivation of *Microcystis Aeruginosa* by peracetic acid combined with ultraviolet: Performance and characteristics, Water Res. 208 (2022) 117847.

[5]	Y. Ren, G. Yu, C. Shi, et al., Majorbio Cloud: A one-stop, comprehensive bioinformatic platform for multiomics analyses, iMeta 1 (2022) e12.

[6]	J. Wang, M. Zhang, S. Hu, et al., Occurrence and cytotoxicity of aliphatic and aromatic halogenated disinfection byproducts in indoor swimming pool water and their incoming tap water, Environ. Sci. Technol. 56 (2022) 17763-17775.

[7]	B. Wu, L. Zhang, S. Wei, et al., Reduction of chromate with UV/diacetyl for the final effluent to be below the discharge limit, J. Hazard. Mater. 389 (2020) 121841.

[8]	R. Li, X. Wu, Z. Han, et al., Removal of antibiotic-resistant bacteria and genes by solar-activated ferrate/peroxymonosulfate: Efficiency in aquaculture wastewater and mechanism, Chem. Eng. J. 474 (2023) 145547.

[9]	G. Li, Z. Xie, Y. Liang, et al., Deep insight into the effect of bicarbonate on pollutant abatement in the UV/chlorine process, Chem. Eng. J. 481 (2024) 148624.

[10]	X. Lu, Y. Chen, H. Chi, et al., Cu(II) assisted peroxymonosulfate for antibiotic resistant bacteria inactivation: a potential disinfection technology in swimming pool, Sci. Total Environ. 876 (2023) 162755.

[11]	C. Ye, K. Zhang, X. Wu, et al., Uncovering novel disinfection mechanisms of solar light/periodate system: The dominance of singlet oxygen and metabolomic insights, J. Hazard. Mater. 443 (2023) 130177.

[12]	E. Wanarska, K.A. Mielko, I. Maliszewska, et al., The oxidative stress and metabolic response of *Acinetobacter baumannii* for aPDT multiple photosensitization, Sci. Rep. 12 (2022) 1913.

[13]	Y. Pan, J.H. Cheng, D.W. Sun, Metabolomic analyses on microbial primary and secondary oxidative stress responses, Compr. Rev. Food Sci. Food Saf. 20 (2021) 5675-5697.

[14]	Y.Y. Pan, D.W. Sun, J.H. Cheng, et al., Insights into inactivation and response mechanisms of sublethal *Listeria monocytogenes* treated by cold plasma with joint transcriptomics and metabolomics, J. Appl. Microbiol. 134 (2023) 1-15.

[15]	X. Yu, X. Jin, J. Tang, et al., Metabolomic analysis and oxidative stress response reveals the toxicity in *Escherichia coli* induced by organophosphate flame retardants tris(2-chloroethyl) phosphate and triphenyl phosphate, Chemosphere 291 (2022) 133125.

[16]	Z. Qi, C. Liu, Metabolic characteristics and markers in viable but nonculturable state of *Pseudomonas aeruginosa* induced by chlorine stress, Environ. Res. 214 (2022) 114111.

[17]	M. Zhang, W. Liu, Q. Qu, et al., Metabolomic modulations in a freshwater microbial community exposed to the fungicide azoxystrobin, J. Environ. Sci. 97 (2020) 102-109.

[18]	S. Nahim-Granados, J.A. Sánchez Pérez, M.I. Polo-Lopez, Effective solar processes in fresh-cut wastewater disinfection: Inactivation of pathogenic *E. coli* O157:H7 and *Salmonella enteritidis*, Catal. Today 313 (2018) 79-85.

[19]	C. Ruales-Lonfat, J.F. Barona, A. Sienkiewicz, et al., Iron oxides semiconductors are efficients for solar water disinfection: A comparison with photo-Fenton processes at neutral pH, Appl. Catal. B-Environ. 166-167 (2015) 497-508.

[20]	W. Wang, H. Wang, G. Li, et al., Catalyst-free activation of persulfate by visible light for water disinfection: Efficiency and mechanisms, Water Res. 157 (2019) 106-118.

[21]	F. Liu, Z. Li, Q. Dong, et al., Catalyst-free periodate activation by solar irradiation for bacterial disinfection: Performance and mechanisms, Environ. Sci. Technol. 56 (2022) 4413-4424.
